# Why sequence the genome of every species? A view from evolutionary biology

**DOI:** 10.1017/S0025315425100556

**Published:** 2025-09-08

**Authors:** Peter Holland

**Affiliations:** Department of Biology, University of Oxford, Oxford, UK

**Keywords:** chromosome, DNA sequencing, evolution, gene duplication, mutation, marine invertebrate

## Abstract

We are in the early stage of a revolution in the field of comparative genomics. Within the past five years, thousands of animal, plant, and fungal genomes have been sequenced and assembled to high quality. There is even serious discussion around sequencing the genomes of every eukaryotic species on earth. Here, I explain why this genomic revolution is happening and discuss the feasibility of sequencing genomes on a massive scale. Having a very wide diversity of genome sequences will accelerate applied research in biomedicine, biotechnology, aquaculture, agriculture, and conservation, and facilitate fundamental research in areas such as ecology, physiology, developmental biology, and evolutionary biology. In this article, I explore new findings and new questions in evolutionary biology emerging from animal genome analyses. Examples are drawn from marine animals such as polychaetes, bivalves, cephalopods, fish, and bryozoans, plus unusual terrestrial groups such as gerbils, moths, and bee-flies. I highlight patterns of mutation, the dynamics of gene families, and chromosomal organisation of genomes as areas ripe for further research. An even wider diversity of genome sequences will be needed to fill the knowledge gaps or investigate emerging puzzles, and a case is made for sequencing the genomes of over 100,000 species.

## Introduction

An initial draft of a human genome sequence was announced in 2000. The UK Prime Minister at the time, Tony Blair, called it ‘the first great technological triumph of the 21st century’ while the US President Bill Clinton said it was ‘the most important, most wondrous map ever produced’ (Office of Science and Technology Policy 2000). When published a year later, the draft sequence was 90% complete with 150,000 gaps, but this already was a resource that would stimulate and facilitate biomedical research and diagnostics, and advance our understanding of human biology (International Human Genome Sequencing Consortium 2001; Venter et al. 2001).

It was also a technological triumph. Just over 20 years earlier, Sanger, Nicklen, and Coulson published a method for determining the sequence of nucleotides – A, C, G, and T – in small fragments of DNA (Sanger et al. 1977). Initially, their dideoxy method used radioactive labelling and could sequence lengths of only around 100 nucleotides in one reaction. Even after 15 years of further development, such as fluorescent dye-labelled dideoxy terminators and capillary gels, it was difficult to push sequencing ‘reads’ beyond 1000 nucleotides. Considering these limitations, it was staggering that the Human Genome Project produced a draft of a genome sequence around three billion nucleotides in length. Now, 25 years after that first published human genome sequence draft, we are in the early phase of a genomic revolution. Sequencing of a single genome today does not draw much attention, although that is unfair since each is an impressive achievement. Sequencing just 90% of a genome would not be considered an acceptable standard today, nor would the fragmentary nature of the original human genome sequence. Instead, most genome sequences published today are ‘chromosomal scale’, with the DNA sequence being of high accuracy, high contiguity, and near completeness.

There is even serious talk of attempting to sequence the complete genome of every species on earth: the ‘Earth BioGenome Project’ (Lewin et al. 2018). The Earth BioGenome Project is a scientific vision. The original paper of Lewin et al. (2018) outlined potential applications and benefits, and also highlighted the huge challenges – technical, practical, financial, and ethical. The vision elicited both excitement and critique but could not be ignored. There were some burning questions. Would it be feasible to sequence the genome of every eukaryotic species on earth (or a large proportion)? What would it cost? Is now the right time? Why should it done? What new biology might be uncovered?

### Why now?

To ask whether now is a good time to consider massive sequencing projects, we need to consider technical aspects. Wider issues such as coordination and cooperation are also important. The cost of DNA sequencing has dropped massively over the past 20 years due to technological advances. This is often presented as ‘cost per million base pairs’, although this refers to ‘raw’ DNA output of a DNA sequencing method and certainly not to a finished genome sequence. These costs dropped by five orders of magnitude between 2001 and 2022 (Wetterstrand 2025). The steepest drop came through the invention of massively parallel DNA sequencing or ‘next generation sequencing’ (NGS). The most widely used of the various NGS methods quickly became the reversible chain termination method developed by Balasubramanian and Klenerman, now known as Illumina DNA sequencing (reviewed by Balasubramanian 2015). This method is remarkable for its enormous output and low cost. A single Illumina run may yield the identity of tens of billions, or hundreds of billions, of nucleotides. For example, in 2025 an individual researcher sending a DNA sample to a commercial DNA sequencing company could receive 30 billion nucleotides of DNA sequence information for just £200 (GBP200; US$260); this equates to under £0.01 (one pence) per million base pairs. By comparison, commercial dideoxy DNA sequencing would cost around £5000 per million base pairs.

The figures are impressive, but surprisingly, the cost per base is a minor reason why now may be ‘the right time’. We need to consider ‘genome assembly’. A genome must be sequenced in pieces and then assembled like a jigsaw. Dideoxy DNA sequencing (also called Sanger sequencing) yields DNA sequences of up to 1400 nucleotides; standard Illumina DNA sequencing yields sequences of just 150 to 300 nucleotides. In both cases, these are tiny. Using such sequences alone would be like attempting a jigsaw with a million pieces, many identical, and with no reference picture.

It is possible to assemble genome sequences using Sanger dideoxy or Illumina technologies but additional costly steps are needed, especially preparation of large insert DNA libraries such as BAC and fosmid libraries propagated in bacteria. This is laborious, technically challenging, and expensive. Sanger sequencing with large insert libraries was used to determine the genome sequence of an ascidian *Ciona intestinalis*, one of the first animal genome sequences published (Dehal et al. 2002): this gave new insights into gene family evolution and confirmed that genes needed for the synthesis and remodelling of the cellulose tunic were acquired by horizontal gene transfer from bacteria or fungi. Similar methods were used to sequence the genomes of two puffer fish species, *Takifugu rubripes* (Aparicio et al. 2002) and *Tetraodon nigroviridis* (Jaillon et al. 2004), giving insight into how evolutionary forces can drive reduction in genome size. Other examples using Sanger dideoxy methodology and large insert libraries include genomes of a burrowing sea anemone *Nematostella vectensis* (Putnam et al. 2007), the Florida amphioxus *Branchiostoma floridae* (Holland et al. 2008; Putnam et al. 2008) and a sponge *Amphimedon queenslandica* (Srivastava et al. 2010), each yielding far-reaching insights into animal genome evolution. With the emergence of NGS technologies, Sanger sequencing was largely sidelined by genome sequencing projects. For example, Illumina sequencing on fosmid libraries was used for the genome of the commercially important Pacific oyster *Magallana gigas* (synonym *Crassostrea gigas*) (Zhang et al. 2012). Illumina was combined with another NGS technology, Roche 454, for sequencing of the genome of the pearl oyster *Pinctada fucata* (Takeuchi et al. 2012).

The big problem with all these examples centres on the additional methods needed, especially making the large insert DNA libraries in bacteria. To simplify the process of genome sequencing, ‘long reads’ were the sought-after breakthrough: these would avoid the need for costly large insert libraries. This leads us to why now is the right time to discuss sequencing all animal genomes. The ability to determine the sequence of long stretches of DNA developed alongside the rise of Illumina sequencing with the emergence of two key technologies: PacBio (developed by Pacific Biosciences, California, USA) and Nanopore (developed by Oxford Nanopore Technologies, ONT, Oxford, UK). Both can read strings of thousands of nucleotides in one stretch (sometimes tens of thousands). However, for over a decade there were two barriers to applying these methods routinely to genome sequencing: high cost and low accuracy. For Nanopore sequencing, both have been improving gradually and ONT can now be used cost-effectively. For PacBio sequencing, there was a sudden improvement around 2018 with the release of PacBio HiFi (high fidelity) technology: in this method, large fragments of DNA are made into circles, which are then sequenced ‘round and round’, producing a consensus. Accurate ‘long reads’ produced by PacBio or ONT can be easily overlapped into ‘contigs’ often a million base pairs long. But often these giant contigs cannot be overlapped with each other, either because of a gap in the sequence information or because of repetitive DNA sequences at their ends.

The second key advance was a clever method to piece together the long contigs into chromosomal-scale ‘scaffolds’ representing the near-complete genome sequence. The method, ‘chromatin capture’ or Hi-C, does not use purified DNA from the species of interest but instead needs isolated nuclei from cells. Using light fixation with formaldehyde, cross-links are formed between DNA regions physically close to each other in the nucleus: this usually means cross-links form within each chromosome, less often between them. Biochemical tricks and Illumina sequencing are then used to decipher which DNA regions were close in the cell, and with this information the large contigs generated by PacBio or ONT can be grouped together into chromosomes.

**Figure 1:**
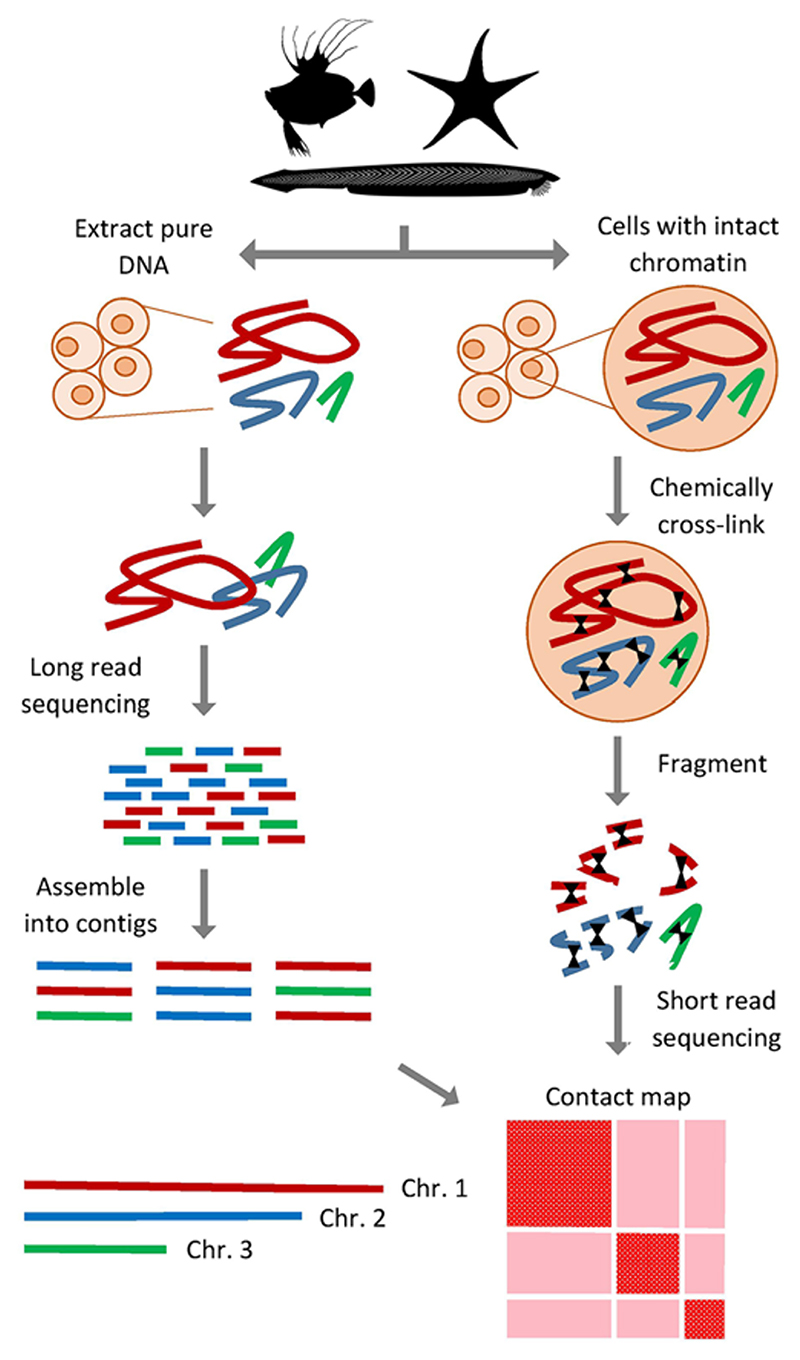
Strategy for genome sequencing and assembly combining long-read DNA sequencing (left-hand flow) and chromatin capture technology such as Hi-C (right-hand flow). Organism outlines from phylopic.org.

By combining these strategies – long-read sequencing with Hi-C – it is now rapid and cost-efficient to produce an extremely high-quality genome sequence from almost any animal (Figure 1). Costs and time-scales vary with scale of operation, with parallelisation offering improved efficiency. For example, the Wellcome Sanger Institute (Hinxton, UK) is currently producing very high-quality animal genome sequences for around £1000 to £5000 (US$1300 to US$6500) per species, at a rate of over 50 species per month. This cost does not include the full cost of collection and identification, undertaken by a network of partners across the ‘Darwin Tree of Life’ network (Darwin Tree of Life Project Consortium 2022). The cost is likely to come down and the rate may increase. Now is therefore the perfect time to discuss the goal of sequencing the genome of every major species in an ecosystem or a country, or even on Earth.

### Why not now?

The initial suggestion for an Earth BioGenome Project (Lewin et al. 2018) elicited positive and negative reactions. One cogent criticism of the original paper focussed on the initial proposal that many genome sequences would be determined in ‘draft’ form using short-read DNA sequencing, and not assembled into high-quality, contiguous assemblies (Robison 2017). Draft genomes cannot be used for studying organisation of genes on chromosomes and may miss genes or features of biological interest. The criticism was valid but is less relevant now because, as outlined earlier, improvements to long-read sequencing and application of Hi-C have made high-quality genome assemblies the norm. This is not to say that genome assemblies being produced in large-scale projects are perfect: they may have small gaps or ambiguities, they ignore chemical modifications to DNA, and most are not ‘T2T’ (telomere-to-telomere) or ‘phased’ (distinguishing the subgenomes from each parent). These limitations affect the detailed analysis of centromeres, telomeres, repetitive DNA, epigenetics, and co-segregated alleles. They are also not ‘pan-genomes’ and instead represent only the genome of one individual. But they are high quality.

A second problem, the difficulty of collecting all species, may have been underestimated. It is hard to assess this critique, but it may have merit considering how difficult it can be to re-collect known species even from well-studied ecosystems such as those across the UK (Lyons 2025). Even so, this is not a reason for abandoning a vision; rather, it could be used to set revised targets; perhaps a goal could be to aim for 150,000 species rather than 1.5 million.

A third criticism is that funds for science could perhaps be better used elsewhere. This is a political and economic argument, not directly a scientific one. Costs and benefits of any scientific project are hard to predict. The cost estimate made by Lewin et al. (2018) for sequencing the genomes of 1.5 million species was around US$4.7 billion including collection costs; as noted, this included draft genomes which are now considered inappropriate. However, a revised cost estimate using long-read data and Hi-C would be in the same order of magnitude, based on sequencing experiences of the Darwin Tree of Life project and including a billion or so for collection costs.

### What use is a genome sequence?

In the original Earth BioGenome Vision, Harris et al. (2018) gave three broad scientific goals for the project. These were (1) ‘Revise and reinvigorate our understanding of biology, ecosystems, and evolution,’ (2) ‘Enable the conservation, protection, and regeneration of biodiversity,’ and (3) ‘Maximize returns to society and human welfare (ecosystem services and biological assets)’. In other words: understand, save, and use.

Under the first goal (‘understand’), genome sequences are already greatly facilitating research in evolutionary biology, developmental biology, physiology, cell biology, neurobiology, ecology, and more. Research uses genome sequences in two distinct ways: directly and indirectly. *Direct* analyses focus on the analysis of the genome sequence itself, usually in comparison to (many) others within a phylogenetic context. This is a particularly useful approach in evolutionary biology. *Indirect* analyses use the genome as a template or reference against which other sequence data are compared (‘mapped’), such as transcriptome reads (bulk or single cell), genomic reads (‘resequencing’), or data from cellular and biochemical analyses (e.g. ATAC-seq, ChIP-seq, CLIP-seq, RIP-seq, CUT&RUN, CUT&Tag, and methyl-seq). These methods can be used, for example, to examine biochemical processes within cells or organisms, to dig deeply into cellular responses to environmental conditions, or to compare cells, individuals, or populations within a species.

The second category (‘save’) highlights the environmental benefits that will come through facilitating genomic-based conservation and breeding strategies. For example, genome sequences can facilitate fisheries management and population evaluation, effective species translocations, and forensic analysis of catch and by-catch (van Oppen et al. 2022). The third stated goal (‘use’) encompasses the human benefits to that will accrue from having access to a diversity of genome sequences. These include (i) improvements to food security through faster genomic selection of farmed species and improved crops, (ii) economic benefits through discovery of new industrial compounds, and (iii) acceleration of pharmaceutical and biomedical research. For example, high-quality reference genomes for oyster species have facilitated discovery of DNA variants associated with disease resistance, growth rate, edibility, and low salinity tolerance (Jiang et al. 2024); these variants are now being used in selective breeding. Similarly, salmon genome sequences have permitted discovery of DNA markers for resistance to sea lice (Correa et al. 2017). In terms of new products, one can easily foresee new pharmaceuticals inspired by genomic analysis of cone snails, nemerteans, or cnidarians, new adhesives from limpets or barnacles, and new antifouling agents targeted to settling marine larvae; in each case, the key will be large numbers of genomes from a diversity of animals.

It is likely these benefits will be enormous in the initial stages. What is unclear is whether the benefits will continue to increase at the same pace as increasing genome numbers. My personal (and imprecise) prediction is that huge economic, societal, and environmental benefits will accrue from sequencing the genomes of the first 100,000–250,000 species. Beyond that, it is possible that benefits may start to plateau as similar genome sequences are determined from closely related species. The implication is that the first £0.5 billion is likely to be a very wise investment indeed. The genomics field is very mindful, however, that much of the world’s biodiversity is found in equatorial regions and the global South, and key issues regarding access and ownership of intellectual property also need addressing.

In the rest of this article, I focus on the new insights and new questions in evolutionary biology emerging from the use and analysis of genome sequences. I primarily use examples from marine animals, but the principles will apply to many groups of organisms.

### Evolutionary biology: pattern and process

Evolutionary biology studies pattern and process. The ‘pattern’ is the evolutionary tree or phylogeny of organisms. Genome sequences hold much information that is useful for inferring the true evolutionary history of organisms. However, except for a minority of ‘difficult’ nodes, it is not necessary to have a high-quality, chromosomal-level genome to infer phylogeny. Lower-cost and lower-quality genome sequences, or even transcriptome data, are obtained more cheaply and can be very useful for this purpose. I do not see phylogeny inference as a primary justification for determining the highest quality genome assemblies. Instead, I will focus on how genome data gives insight into the ‘processes’ underpinning evolution.

Development, anatomy, and physiology change over evolutionary time because of inherited changes in DNA sequence. This is not to imply that there are simple rules between genotype and phenotype. RNA and protein products from genes interact in complex ways giving emergent properties that are difficult to predict. Environmental conditions alter how, when, and to what extent genes are expressed – and how their products interact – modulating the phenotype produced. Even so, ultimately it is inherited changes in the genome that gave rise to the diversity of life on earth, that facilitate adaptation, and that underpins much of the variation found within populations.

What causes these genetic differences between or within species? This is usually seen as a two-stage process – random mutation followed by selection or drift – although I will argue that this can be over-simplistic. First, consider mutations. These include single-nucleotide substitutions, some arising from damage to DNA that is not repaired correctly, others caused by mis-incorporation of nucleotides during DNA synthesis. There are also larger-scale changes such as tandem gene duplications, some caused by mispairing of chromosomes at meiosis. Chromosomes can fuse, break, or get internal rearrangements. Mobile DNA elements, or their reverse transcriptase products, can cause copying of genes or exons, or disruptions to regulatory elements. Polyploidy events generate duplications of the whole genome, and new loci can arise by occasional ‘horizontal’ transfer of genes between species possibly mediated by viruses. All these events result in genetic polymorphism within species. The second stage is selection or drift, causing changes of frequency of polymorphisms and ultimately fixation in populations. This sounds straightforward, but interpreting the differences between species is complicated.

### Evolutionary biology: finding the differences that matter

If we wish to understand the ‘process’ of evolution, we must solve the puzzle of which genetic differences influence which phenotypic differences. When studying adaptive differences between populations within one species, it is logically straightforward (though technically demanding) to find these associations using genomics. In brief, one searches for DNA variants that co-segregate with the phenotypic trait of interest, either in the wild or in controlled crosses. The approach does not need fully assembled genomes from every individual. Usually, just one high-quality ‘reference genome’ is determined, and then short read genomic information (e.g. from Illumina sequencing) from multiple individuals is compared against the reference. The approach has proven spectacularly successful. Excellent examples are provided by studies on the three-spined stickleback *Gasterosteus aculeatus*, a widespread coastal marine fish which invaded freshwater habitats in the Northern Hemisphere after retreat of the ice sheets (Reid et al. 2021). Marine and freshwater habitats pose different selective pressures and natural selection on genetic variants drove local adaptation. Mutations have been identified which associate with phenotypic differences such as size of pelvic and dorsal spines, extent of defensive bony lateral plates, and pigmentation (Roberts Kingman et al. 2021). Our understanding of evolutionary biology has been advanced greatly through this approach, with new insights into questions such as the relative frequency of non-coding versus coding sequence variation under natural selection (Jones et al. 2012), the importance of standing genetic variation in populations (Reid et al. 2021; Roberts Kingman et al. 2021), and, in mammals and insects, the role of hybridisation and introgression in moving pheno-typic traits between species (Jones et al. 2018; Thawornwattana et al. 2023).

Comparing between species raises bigger hurdles. The problem is simply that distant species have thousands of genomic differences and a swathe of phenotypic differences. We may find extra genes in one species, a chromosomal rearrangement, or a change in a gene sequence. We may even be able to detect that these changes have been subject to selection. But are they linked to specific phenotypic changes? When the biochemical function of a protein is known, this is feasible. Good examples are provided by opsin genes which encode light-receptive proteins used in the retina, each opsin protein tuned to a different optimal frequency of light which can be determined experimentally. For example, changes in opsin gene sequences generated blue-shifted light receptors adapted to deep-water vision in whales and sea snakes (Dungan and Chang 2022; Seiko et al. 2020).

Usually, however, it is very hard to track down the functional consequences of genomic differences. We may make plausible hypotheses based on which species have which genes or genomic features, but testing these ideas is difficult. Furthermore, when comparing species we must also factor in unexpected findings and puzzles emerging from comparative genomics. Findings that remind us that we have much still to learn about how genomes evolve. I will look at just three of these in detail using animal examples: nucleotide composition, gene duplication, and the organisation of genes on chromosomes.

### Evolutionary puzzles: is mutation biased?

As more genome sequences have been determined, some puzzling oddities have emerged, raising questions and challenging simplistic views of how evolution works. Some findings have highlighted that mutation is not evenly distributed across genomes or across the diversity of life, suggesting that not all genes or all traits are equally labile in evolution. Mutations may be random in terms of how they relate to function, but this does not imply that all genes or species have an equal probability of mutation. This is well illustrated by considering base composition.

The DNA double helix is built from four nucleotides: A, C, G, and T. Due to complementary base pairing, the number of A nucleotides will equal the number of T nucleotides, and C will equal G, but what about the ratio of A:T pairs to G:C pairs? A survey of almost 2000 animal genomes found a sample average of 60% AT and 40% GC (Kyriacou et al. 2024), but there are deviations that pose questions. Two striking patterns have emerged that still demand full explanation. First, there are species that are GC-rich or AT-rich across the whole genome. Second, there are genomes with ‘peaks’ of GC-rich DNA in an otherwise normal chromosomal background.

**Figure 2:**
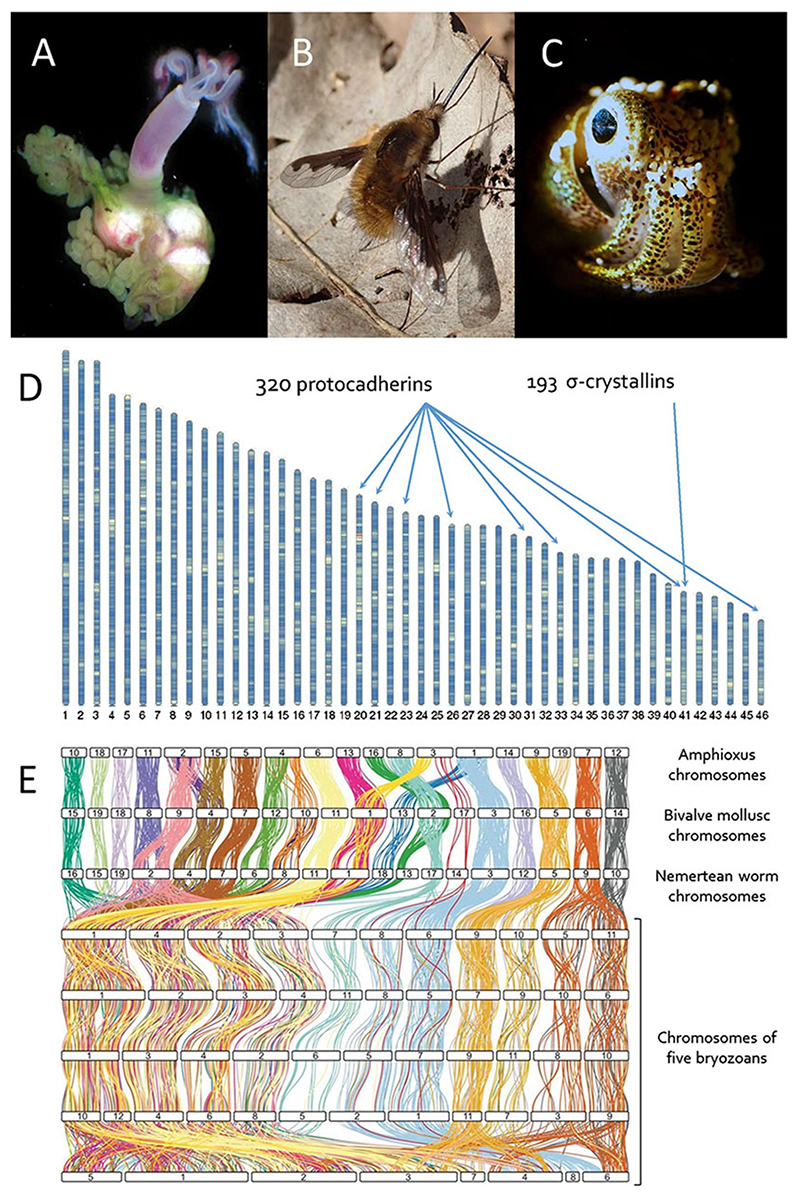
Unusual genome evolution. (A) Polychaete *Osedax frankpressi* has an AT-rich genome. Photo: Greg Rouse. (B) Bee-fly *Bombylius major* has an AT-rich genome. Photo: Liam Crowley. (C) Cephalopod *Euprymna berryi* has arrays of duplicated genes. Photo: Ryuta Nakajima. (D) Chromosomes of *Euprymna* showing locations of duplicated protocadherin and σ-crystallin genes. From Gavriouchkina et al. (2025), modified from the original and used under CC BY 4.0. (E) Ribbon plots comparing homologous genes between chromosomes reveal remarkable conservation of macrosynteny between divergent phyla but extensive scrambling in Bryozoa. Top to bottom: *Branchiostoma floridae, Pecten maximus, Lineus longissimus, Membranipora membranacea, Bugulina stolonifera, Watersipora subatra, Cryptosula pallasiana*, and *Cristatella mucedo*. From Lewin et al. (2025), modified from the original and used under CC BY 4.0; copyright Cold Spring Harbor Laboratory Press.

In the first category, examples have been discovered amongst marine animals. For example, the deep-sea polychaete *Osedax frankpressi*, which lives attached to the bones of dead whales, has a genome GC content of just 29% (hence AT content 71%; Moggioli et al. 2023; Figure 2A). This is not a general feature of marine animals or even polychaetes; the closely related Vestimentifera have a more ‘normal’ GC of over 40%. There are also high GC marine animals such as sea lamprey *Petromyzon marinus* (46% GC; Smith et al. 2013) and low GC terrestrial insects such as bee-flies *Bombylius* sp. (26% GC; Kyriacou et al. 2024; Figure 2B). It seems surprising that an animal genome can have its vast DNA double helices made up of three-quarters A:T pairs and only one-quarter G:C pairs. In most cases, these genome-wide shifts in GC content do not seem to relate to ecological conditions and are unlikely to be adaptive. The *Osedax* and *Bombylius* studies give clues to what might drive these patterns. In *Bombylius* (Figure 2B), the whole genome averages 26% GC, but protein-coding genes are less extreme at 31% while the third position of each codon is more extreme at 13% GC. Third positions of codons can often change without affecting protein sequence, whereas first and second positions change alter protein sequence. This suggests that a widespread mutational bias towards A and T has been occurring in this evolutionary lineage, these changes being permitted when neutral (or slightly deleterious) but resisted by natural selection at sites where mutations are strongly deleterious. It is known that eukaryotes have a bias in mutational frequency towards A and T, because the commonest forms of DNA damage are spontaneous deamination of C or methyl-C bases leading to U:G or T:G pairs. These altered bases can lead to C:G to T:A mutations if not recognised and corrected by the Base Excision Repair (BER) process (Krokan and Bjørås 2013). In the polychaete *Osedax frank-pressi*, some of the conserved BER genes have actually been lost in evolution, perhaps explaining the genome-wide drift towards A:T pairs (Moggioli et al. 2023). These phenomena, however, raise questions. How were such functionally important gene losses permitted in evolution? Was the gene loss a consequence of genetic drift in small populations? Has this occurred in other animal groups? And have they increased the probability of slightly deleterious mutations, spread across the genome, becoming fixed in populations?

Genomes with ‘peaks’ of GC-rich DNA that deviate from the rest of the genome also raise questions. The most extreme examples known come from birds and a group of mammals, the gerbils (Hargreaves et al. 2017; Hron et al. 2015); no comparable cases have been found so far in marine animals. In gerbils, one of the large GC-rich peaks encompasses genes with fixed deleterious mutations caused by GC-biased codon changes (Dai et al. 2020; Hargreaves et al. 2017). These are dramatic non-adaptive changes that compromise the function of key proteins. The peaks of GC are thought to be driven by a meiotic phenomenon called GC-biased gene conversion (gBGC). In brief, if a polymorphic site lies close to a site of meiotic recombination, the four gametes may not inherit the two alleles in a Mendelian ratio, but instead A:T alleles can be replaced by G:C alleles (Dai et al. 2020). Regardless of their adaptive significance, the G:C alleles can therefore spread in a population. This is usually a minor effect, as the sites of meiotic recombination on chromosomes change each meiosis; however, hotspots of meiotic recombination have become stabilised in birds and gerbils. Once again, this leads to important questions. If gBGC can distort Mendelian inheritance dramatically, this could skew the direction of evolution through the spread of deleterious alleles. In gerbils, this effect likely caused predisposition to type II diabetes and metabolic constraints. Are gerbils and birds really the only examples in animals, or will additional cases be found amongst the vast diversity of marine taxa? More genomes need to be analysed to answer these questions.

### Evolutionary puzzles: are gene duplications always adaptive?

When the first animal genome was sequenced, from the nematode *Caenorhabditis elegans*, a striking finding was the discovery of over 200 genes encoding nuclear hormone receptors (Clarke and Berg 1998). Gene duplication, leading to ‘families’ of genes, had long been studied, but few expected a well-studied gene family to have such a hidden repertoire. As more and more genomes were sequenced, expansions of other gene families were found. For example, genome sequencing of the cephalopods *Octopus bimaculoides* and *Euprymna berryi* revealed 168 and 320 genes encoding protocadherin proteins involved in cell-cell adhesion and recognition (Albertin et al. 2015; Gavriouchkina et al. 2025; Figure 2C, D), and a genome sequence of the Pacific oyster *Magallana gigas* (synonym *Crassostrea gigas*) had 88 *heat shock protein 70* (*HSP70*) genes and 136 *cytochrome P450* (*CYP450*) genes (Zhang et al. 2012). *Euprymna berryi* also has a remarkable 193 genes encoding cephalopod-specific glutathione-*S*-transferase-derived proteins (Gavriouchkina et al. 2025; Figure 2D). These extensive gene duplication events are probably adaptive, with a large functional diversity of protocadherin proteins aiding development of the complex *Octopus* or *Euprymna* brain, glutathione-*S*-transferase-derived proteins deployed as σ-crystallins used in formation of the non-cellular cephalopod lens, and large numbers of *HSP70* and *CYP450* genes in oyster aiding responses to environmental stressors in the intertidal zone.

As each new genome is sequenced, it is common for analyses to focus on gene family expansions. It is equally common to see speculation about how the extra genes are related to the ecology or anatomy of a species. No doubt many of the cases discovered are indeed adaptive. There is need for some caution, however. First, we do not fully understand when gene family expansions are driven primarily by differential mutation (do some genes duplicate more?) or when the key factor is differential retention (do all genes duplicate but only some are retained by natural selection?). Classical evolutionary biology puts emphasis on selection rather than mutation: under this view, gene duplications imply that selection favoured the extra genes. However, recent studies in nonmarine species point to exceptions: in moths huge duplications of a Hox gene in some species seem to have been driven by the vagaries of transposable elements facilitating tandem duplication mutations probably with a neutral effect (Mulhair et al. 2023). The second reason for caution is that in early genome studies the species sampling was low so that gene duplications could not be mapped onto a phylogeny. Without good comparative data, identifying adaptive reasons for diversification of gene families is imprecise. Third, it is becoming apparent that some gene families can be highly dynamic. For example, comparison between octopus and squid protocadherin genes suggests extensive independent duplication (Albertin et al. 2015; Gavriouchkina et al. 2025). At first sight, this may seem surprising since nervous system complexity was a characteristic present in their common ancestor. The conundrum can be solved by viewing gene family evolution as a balance between gene birth and death; when we compare a few living species, we are seeing only the tips of the distribution. With genomes only from distantly related species, we cannot hope to reconstruct complex gene dynamics. This dynamic nature is not true for all gene families, however, and many smaller gene families seem static in evolution. These could be sites less prone to duplication, or more likely they are loci at which any duplication is deleterious. In general, if we wish to understand how and when large gene families diversified, and how duplicated genes are recruited to new roles in evolution, we often require the complete genome sequences from large numbers of species. Sporadic sampling will not necessarily work.

Perhaps the most dramatic mode of gene duplication occurs through polyploidy or ‘whole genome duplication’ (WGD). Many ancient WGD events occurred during the evolution of plants, but WGD seems rarer in animals. Nonetheless, WGD events occurred during the early evolution of vertebrates, then again on the lineage leading to teleost fish, in salmonid fish, in cyprinid fish, and in the sturgeon/paddlefish clade. Within invertebrates, WGD occurred in caenogastropods, including cone snails, in stylommatophoran land snails, in bdelloid rotifers, and in horseshoe crabs, spiders, and scorpions (reviewed by Au et al. 2025). In every case, genomic data provided the definitive proof that these were indeed ancient WGD events. For example, comparison of the genomes of amphioxus *Branchiostoma floridae* and humans revealed ‘quadruple conserved synteny’, whereby regions of the amphioxus genome can be aligned to four regions of human reflecting two WGD events in early vertebrate evolution (Putnam et al. 2008; Simakov et al. 2020). Similarly, the human genome has ‘double conserved synteny’ with pufferfish genomes reflecting an additional WGD in teleost fish evolution (Jaillon et al. 2004). Other mechanisms of gene duplications, such as tandem duplication and retrogene formation, leave very different genomic signatures from those left by WGD; these can also be traced by analysis of synteny.

How important were WGD events in animal evolution? It is certainly an attractive idea that WGD facilitates evolutionary change. One suggestion is that WGD enables duplication of interacting sets of genes, maintaining stoichiometry of interacting molecules, or providing gene network robustness: whole genetic pathways can then diverge to effect new roles (Carretero-Paulet and de Peer 2020). Another hypothesis focuses on how neutral changes can lead to speciation. After WGD, the two sets of chromosomes will have redundant genes. If redundant genes are lost stochastically from each of the duplicated chromosomes, this could quickly make different populations genetically incompatible with each other, accelerating speciation but without immediate adaptive change (Lynch and Force 2000). Other models have also been discussed (Van de Peer et al. 2009): a challenge is how to test these. Examining the timing of adaptive radiation or speciation following WGD is not a reliable test, since it can take many millions of years for four identical chromosomes to gradually resolve into two distinct chromosome pairs permitting functional divergence; this can even occur at different rates on different chromosomes (Martin and Holland 2014; Robertson et al. 2017). A better test may be to ask whether WGD-derived gene duplications are preferentially used in novel, lineage-specific roles, compared to genes duplicated by other routes.

In summary, genome analyses have revealed a complex interplay of factors shaping multigene families. Tandem gene duplication is probably commoner than often appreciated and balanced by dynamic gene loss; in any given species, we only see a snapshot of a dynamic process. At some chromosomal locations, or in some lineages, gene duplication mutations may also be accelerated by changes to transposable elements, rapidly generating arrays of genes sometimes of limited functional relevance. In other cases, selection may fix new genes and deploy them for new roles. Hence, mutation, selection, and drift must all be considered – with much still to be learnt about mutation – and many genomes are required to pull apart these influences. Whole genome duplication must also be probed further using additional genomes and functional analyses. Key questions include what factors influence retention or loss of genes following WGD, what permits persistent of polyploids after their generation, how chromosomes undergo reversion to preferential pairing (rediploidisation), and whether WGD-derived duplications differ from single gene duplications in evolution.

### Evolutionary puzzles: do genes care who their neighbours are?

A traditional view sees eukaryotic genes as evolutionary nomads, able to function wherever they are on whichever chromosome. Since eukaryotic genes generally have their own promoters and enhancers, and transcription factors diffuse within the nucleus, it is reasonable to think that gene position does not matter very much. Exceptions are known, such as globin genes and Hox genes, where clustered genes are regulated through a shared mechanism and there is selection pressure to maintain gene neighbours (Duboule 2022). Even here, the genes have split apart in some evolutionary lineages, presumably as the selection pressure is relaxed (Ferrier and Holland 2002). With so few cases, the emerging picture for many years was one in which very few genes ‘care’ about their neighbours, the rest being dotted around the genome in a random arrangement that could be shuffled in evolution with no phenotypic consequence.

As more animal genomes were sequenced, this picture became more complex. Perhaps the main conclusion emerging is that we know little about the evolutionary forces that shape the location of genes on chromosomes. On the one hand, there is microsynteny, where neighbouring ‘sets’ of genes seem to be maintained by selection. Few examples are known so far, beyond globin and Hox genes, with an interesting case being a set of ‘pharynx-expressed’ genes in hemichordates and chordates, maintained in close proximity across deuterostome evolution (Simakov et al. 2015). Even more puzzling is macrosynteny: conservation of genes at a chromosomal level. Small-scale rearrangements and lineage-specific genes are ignored in the analysis of macrosynteny, permitting chromosomes to be compared between very diverse animals. A remarkable picture emerges. It turns out that animals as diverse as hemichordates, amphioxus, echinoderms, polychaete worms, and bivalve molluscs, and to some extent even cnidarians and sponges, have very similar chromosomes. To most zoologists, this discovery was very unexpected. For example, chromosome 3 (the third largest chromosome) of the scallop *Pecten maximus* has essentially the same genes as chromosome 3 of the nemertean *Lineus longissimus*, chromosome 1 of amphioxus *Branchiostoma floridae*, chromosome 3 of the peanut worm *Sipunculus nudus*, or chromosome 5 of the polychaete *Owenia fusiformis* (Martín-Zamora et al. 2023; Lewin et al. 2024, 2025; Figure 2E). This means large numbers of genes have stayed together for hundreds of millions of years. There have been occasional fusions or fissions; for example, *Owenia* chromosome 1 is equivalent to *Pecten* 4, 16, and 18 (Lewin et al. 2024, 2025; Martín-Zamora et al. 2023). The chromosomal compositions are so comparable between phyla that it is even possible to deduce the ‘ancestral linkage groups’ of long extinct ancestors. In other words, we can say how many chromosomes were present in an animal living half a billion years ago and which genes were on each chromosome. We do not know why genes have stayed remarkably faithful to their chromosomes. Perhaps there are unknown selective reasons such as shared regulation of linked genes, maintenance of linkage between co-adapted alleles, or selection against fissions and fusions to retain an optimal number of chromosomes for effective cell division or recombination.

There are groups of animals where these rules have broken down. For example, the long-conserved gene linkages have been massively scrambled in Clitellata (leeches and earthworms), Tunicata, Bryozoa, and Brachiopoda (Lewin et al. 2024, 2025; Figure 2E). Not only have chromosomes fused and broken, but in each case the genes were shuffled amongst each other extensively. These rearrangements can occasionally be used as phylogenetic markers, for example, supporting a close relationship between Bryozoa and Brachiopoda (Lewin et al. 2025). Of more general interest are the underlying causes. If maintenance of ancestral linkage groups was selectively advantageous for hundreds of millions of years in some animals, then why has radical restructuring been permitted in other evolutionary lineages? Whatever the reasons, this shuffling will have brought new combinations of genes together, potentially permitting new phenotypes through changes to gene regulation. Much work is required to untangle these events, map their frequency, and understand their relevance to phenotypic evolution.

## Conclusions

The first animal genome sequence was published in 1998 and a draft human genome announced to the world soon after, but for the next 20 years, the technology for DNA sequencing and genome assembly was still in its infancy. Important projects were completed in this period through the perseverance of committed research consortia. These early genome sequences included marine animals such as sea anemone, sponges, placozoan, ctenophore, sea urchin, tunicates, amphioxus, hemichordate, bivalves, cephalopods, polychaetes, and more. But these projects needed large insert DNA libraries to be constructed before DNA sequencing: a costly and laborious process. I have explained earlier that a step-change in our ability to sequence genomes occurred between 2018 and 2020, with the widespread adoption of long-read DNA sequencing and chromatin capture methods enabling genomes to be sequenced more rapidly and cost-effectively. This caused a shift in mindset. ‘Single species’ projects are no longer viable; projects can now be focussed on sequencing the genomes of hundreds of related species, or thousands of species in an ecosystem or geographic region. But, at present, sequencing of a million animal species is a vision not a project.

Why sequence all these genomes? The riches hidden in genomes are immense. There is excitement over the application of genomic data in conservation, fisheries, and aquaculture, and of course for the discovery of new biomedicines and new industrial compounds. Genomic biology will certainly accelerate such applied research. My personal view, as noted earlier, is that we will need to seek a realistic balance between investment in numbers of genomes sequenced and the pace of discovery science. For applied research, a target of 100,000–250,000 species may be a realistic target, rather than aiming for the genome sequence of every species on earth.

One goal of this article was to consider how a wealth of high-quality animal genome sequences might accelerate evolutionary biology research. I offer seven suggestions. *First*, phylogenetic inference is not the primary argument for generating chromosomal-level animal genome assemblies. Occasionally, a feature of genome organisation can be a useful phylogenetic marker, but usually lower-quality genomic data, or even transcriptomic data, will be sufficient for obtaining thousands of phylogenetically informative gene sequences. *Second*, chromosomal-level genomes greatly facilitate the study of intraspecific variation; for example, when searching for loci underpinning adaptive traits. Such research is already yielding surprising insights into how adaptation works at the molecular level, revealing the importance of both cis-regulatory and coding sequence mutations, and highlighting the power of inter-species hybridisation. *Third*, finding the genetic basis for differences between higher taxonomic ranks (e.g. classes or phyla) is difficult, even with genomic data. Here, there is a strong argument for sequencing large numbers of species to ascertain precisely when genomic characters (such as new genes) arose in relation to phylogeny. *Fourth*, although mutation is core to evolutionary change, our understanding of mutational mechanisms and frequencies is inadequate. A wider diversity of high-quality genome sequences are clearly needed to help fill this knowledge gap. Lessons from the unusual genomes of the deep-sea polychaete *Osedax* and the bee-fly *Bombylius* are salutary. *Fifth*, research has revealed gene families to be surprisingly dynamic, often with recurrent duplication and loss. Relating these patterns to the formation of new genes – and new biological traits – demands genomic data from many more species. New genes were likely critical in the evolution of new body plans and new biology, so they should be fully explored. *Sixth*, molecular evolution is more complex than the interplay of mutation, selection, and drift; for example, gBGC can bias inheritance and influence fixation of alleles. Again, more genomes are required to assess the generality of this phenomenon. *Finally*, what about genome organisation? The advent of chromosomal-scale genomics has revealed astonishing patterns of chromosome evolution across animal evolution, such as maintenance of chromosomes for hundreds of millions of years. Other surprises are sure to await a deeper dive into animal genomes; for this, investigating the vast diversity of marine taxa will be key.

## Data Availability

No data are associated with this article.
